# Supplementary material for: Highly accurate genome assembly of an improved high-yielding silkworm strain, Nichi01
Source: G3 (Bethesda). 2023 Feb 23;13(4):jkad044. doi: 10.1093/g3journal/jkad044 (PMC10085791; doi:10.1093/g3journal/jkad044)
Supplement: jkad044_Supplementary_Data [file jkad044_supplementary_data.zip › Waizumi_et_al._supplementary_fig.pdf]

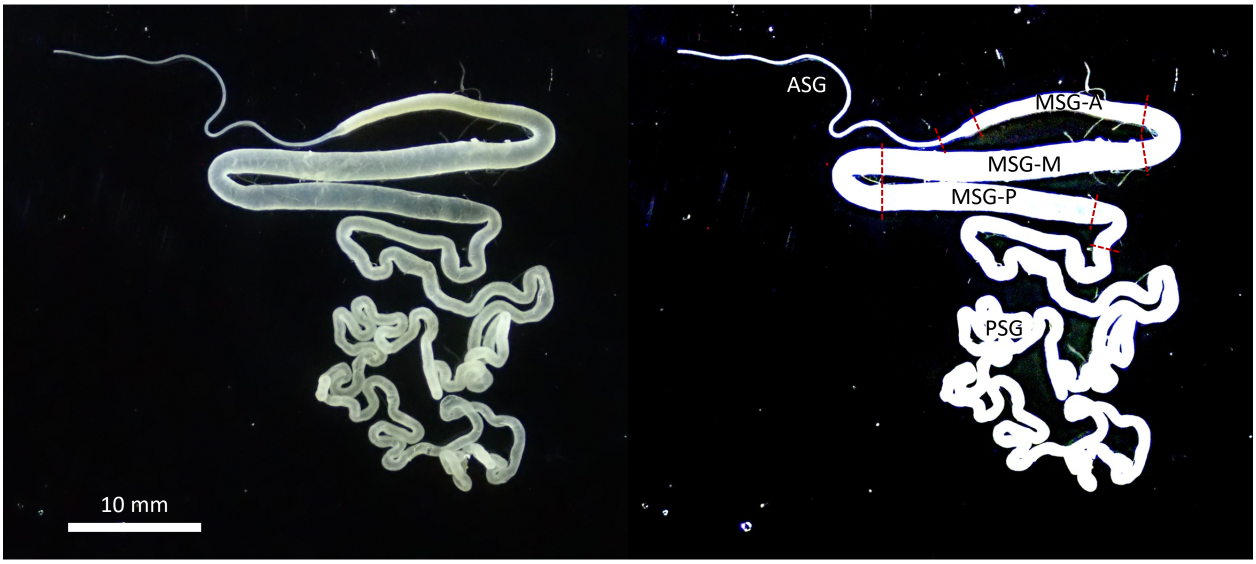

Supplementary Figure S1. Silk gland of Nichi01 fifth-day final instar larva.

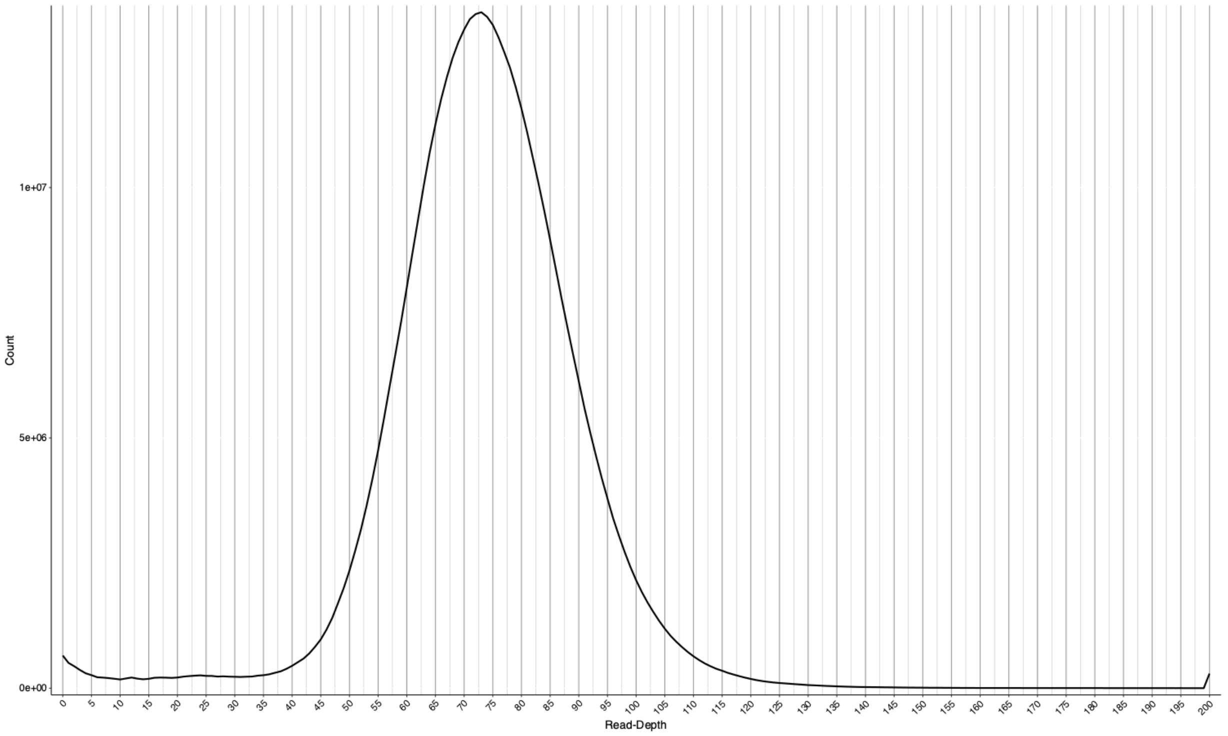

Supplementary Figure S2. Coverage histogram of the PacBio long reads on the corrected contigs.

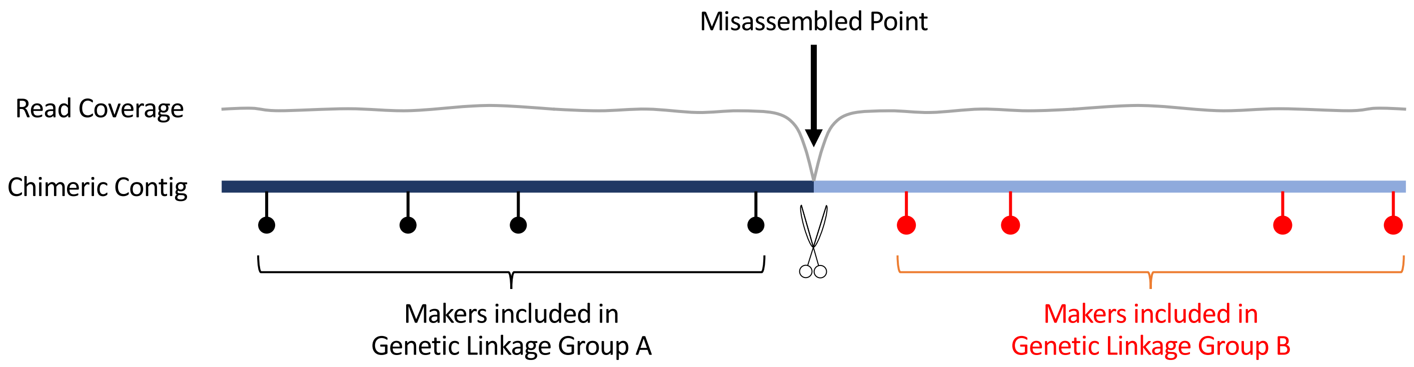

Supplementary Figure S3. Explanatory drawing of misassembly correction.

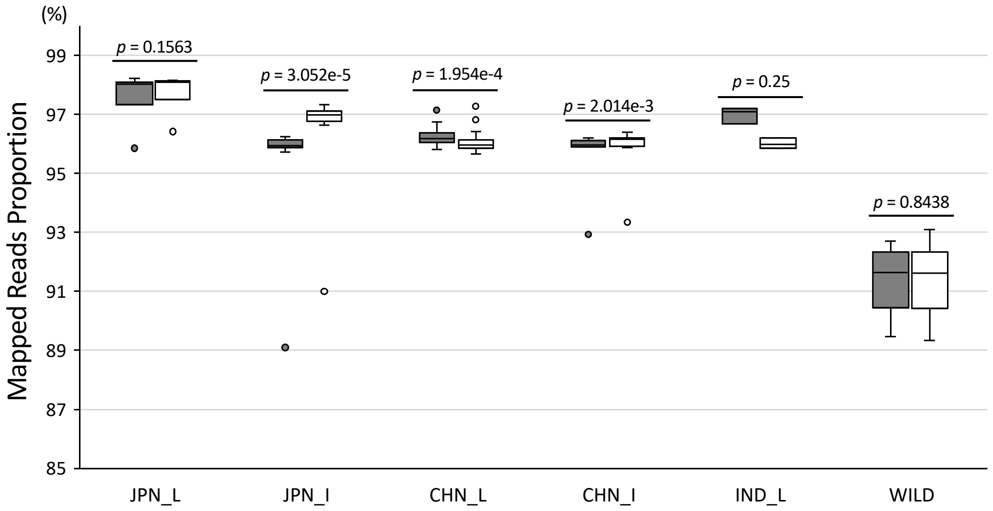

Supplementary Figure S4. Proportion of mapped genomic short reads.
