# Supplementary figures and images for: Highly accurate genome assembly of an improved high-yielding silkworm strain, Nichi01
Source: G3 (Bethesda). 2023 Feb 23;13(4):jkad044. doi: 10.1093/g3journal/jkad044 (PMC10085791; doi:10.1093/g3journal/jkad044)

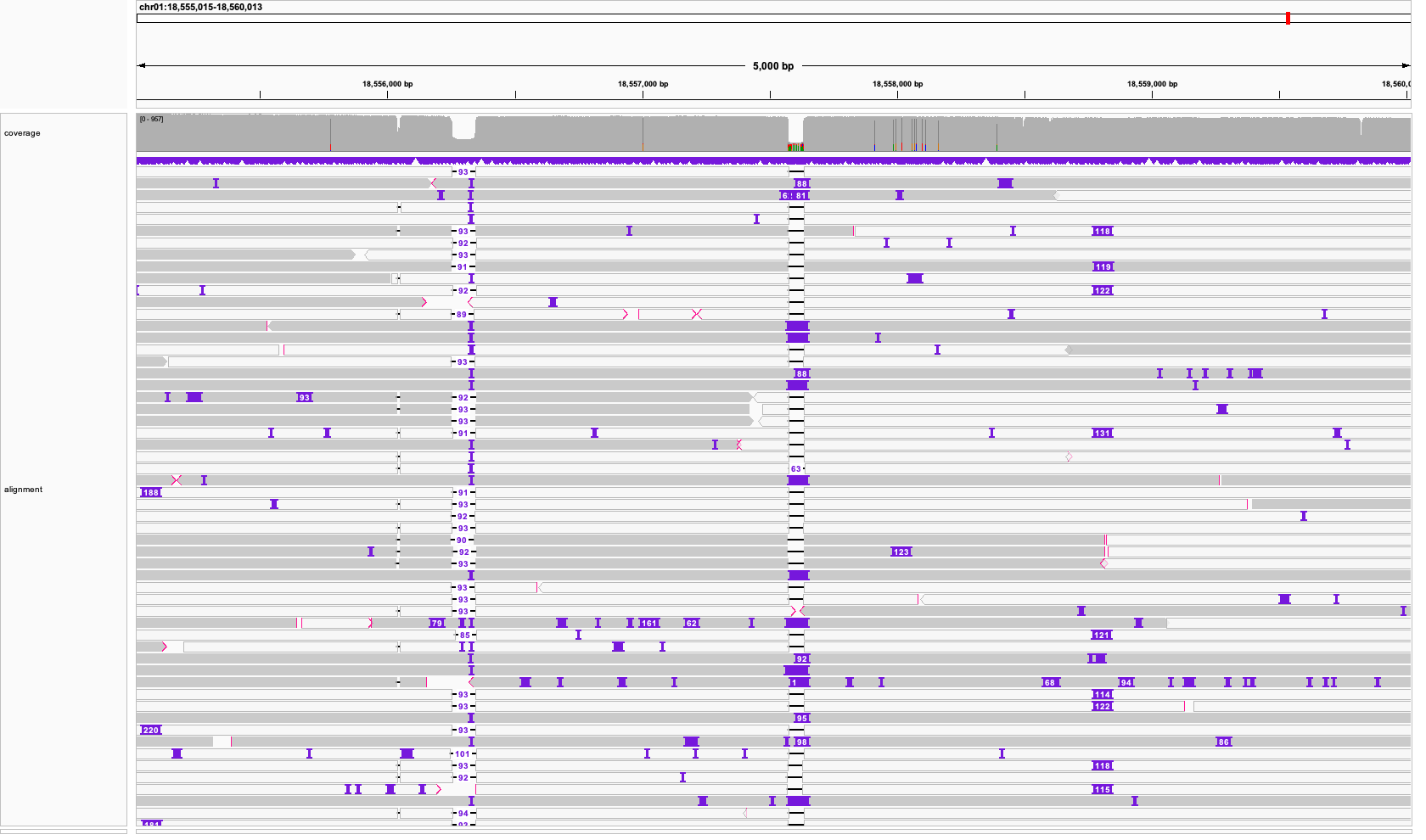

Supplement: jkad044_Supplementary_Data [file jkad044_supplementary_data.zip › Supplementary_Image/chr01.png]

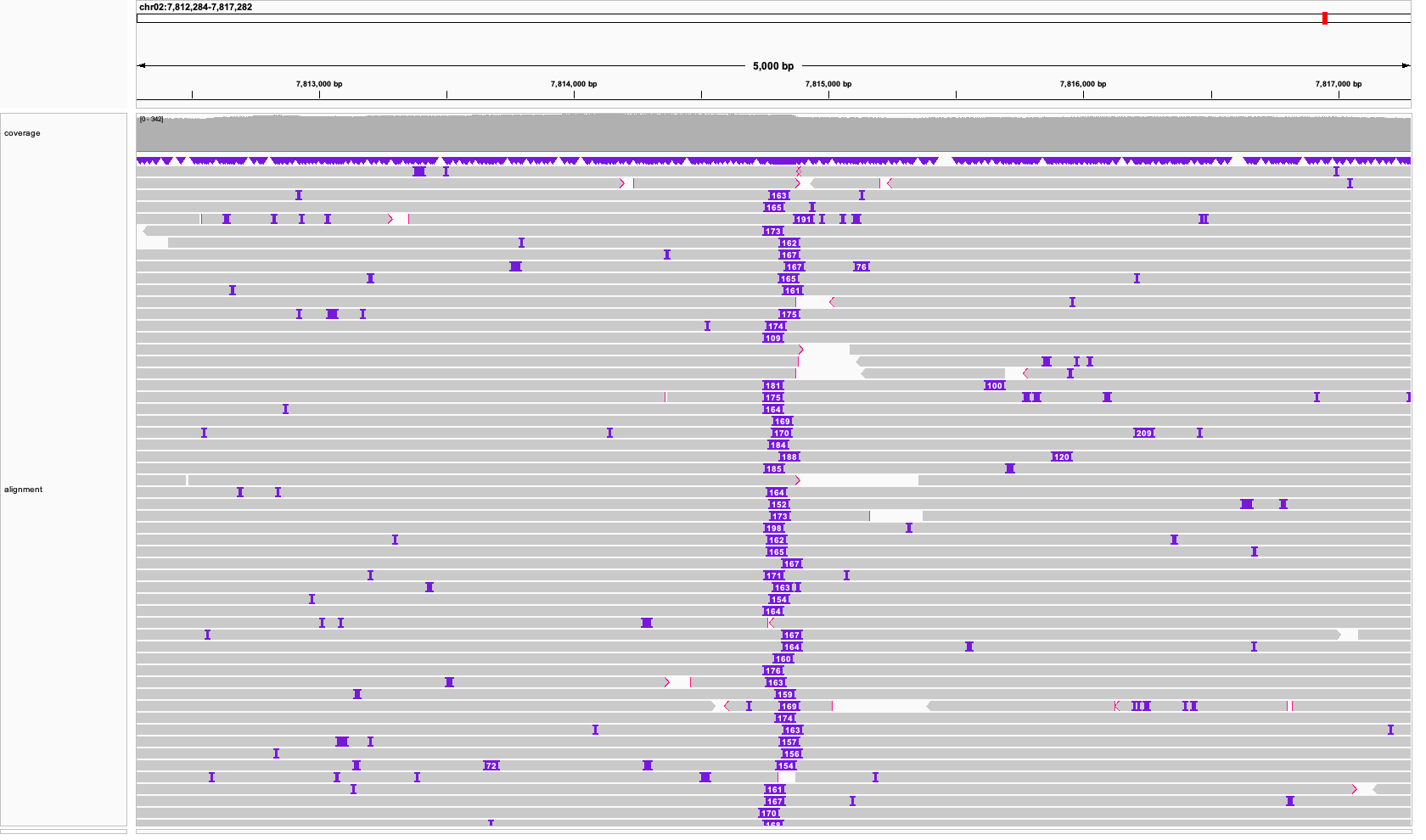

Supplement: jkad044_Supplementary_Data [file jkad044_supplementary_data.zip › Supplementary_Image/chr02.png]

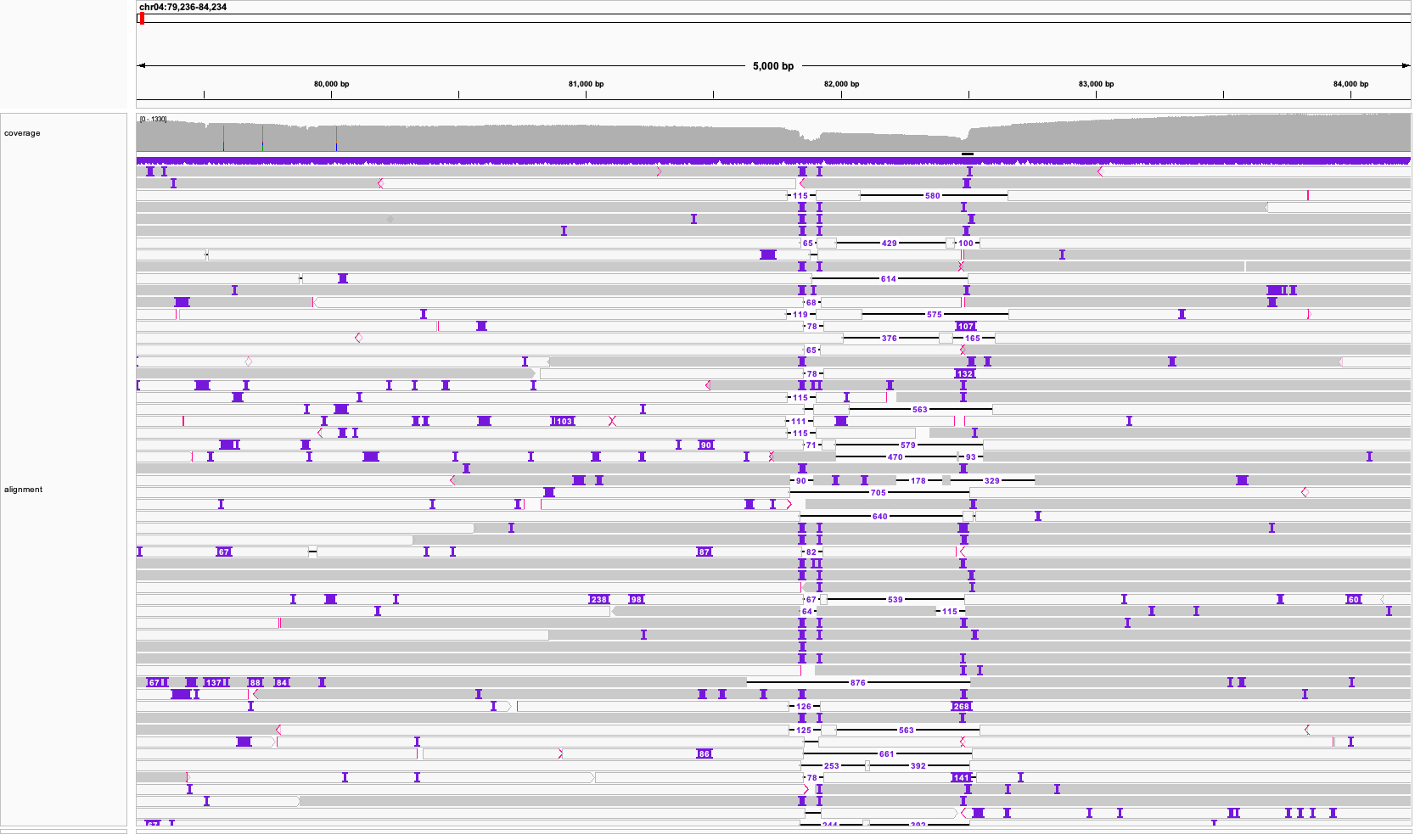

Supplement: jkad044_Supplementary_Data [file jkad044_supplementary_data.zip › Supplementary_Image/chr04.png]

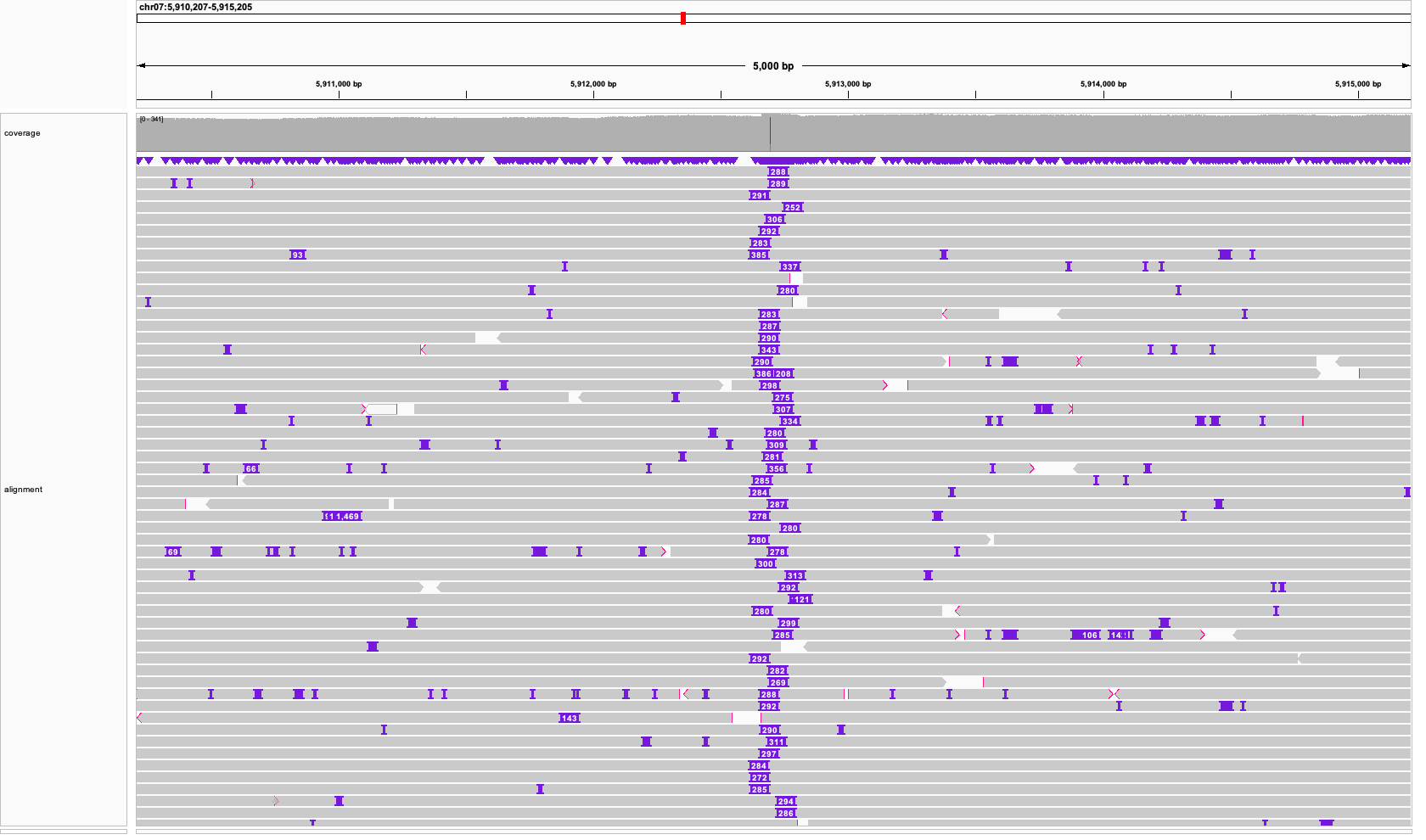

Supplement: jkad044_Supplementary_Data [file jkad044_supplementary_data.zip › Supplementary_Image/chr07.png]

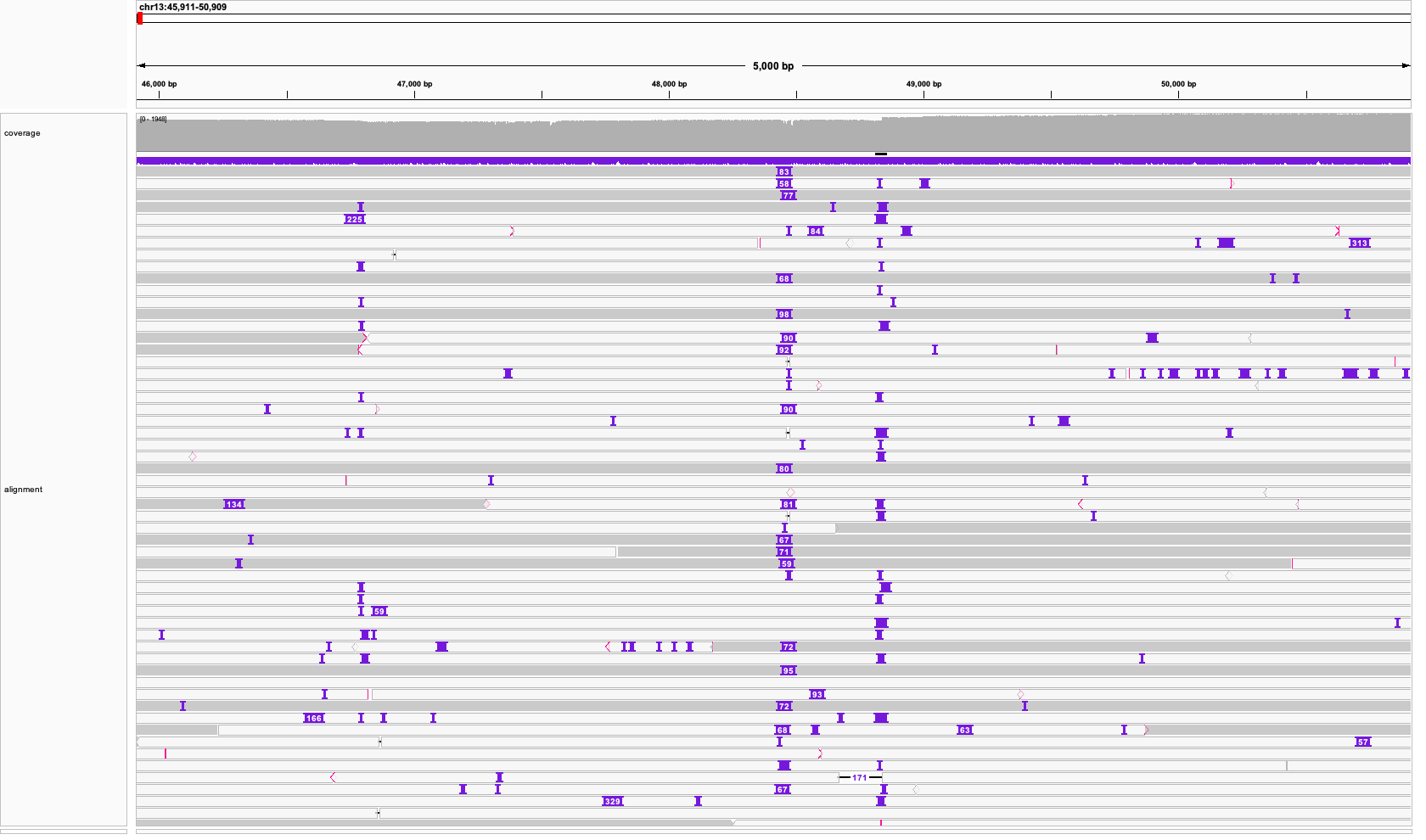

Supplement: jkad044_Supplementary_Data [file jkad044_supplementary_data.zip › Supplementary_Image/chr13.png]

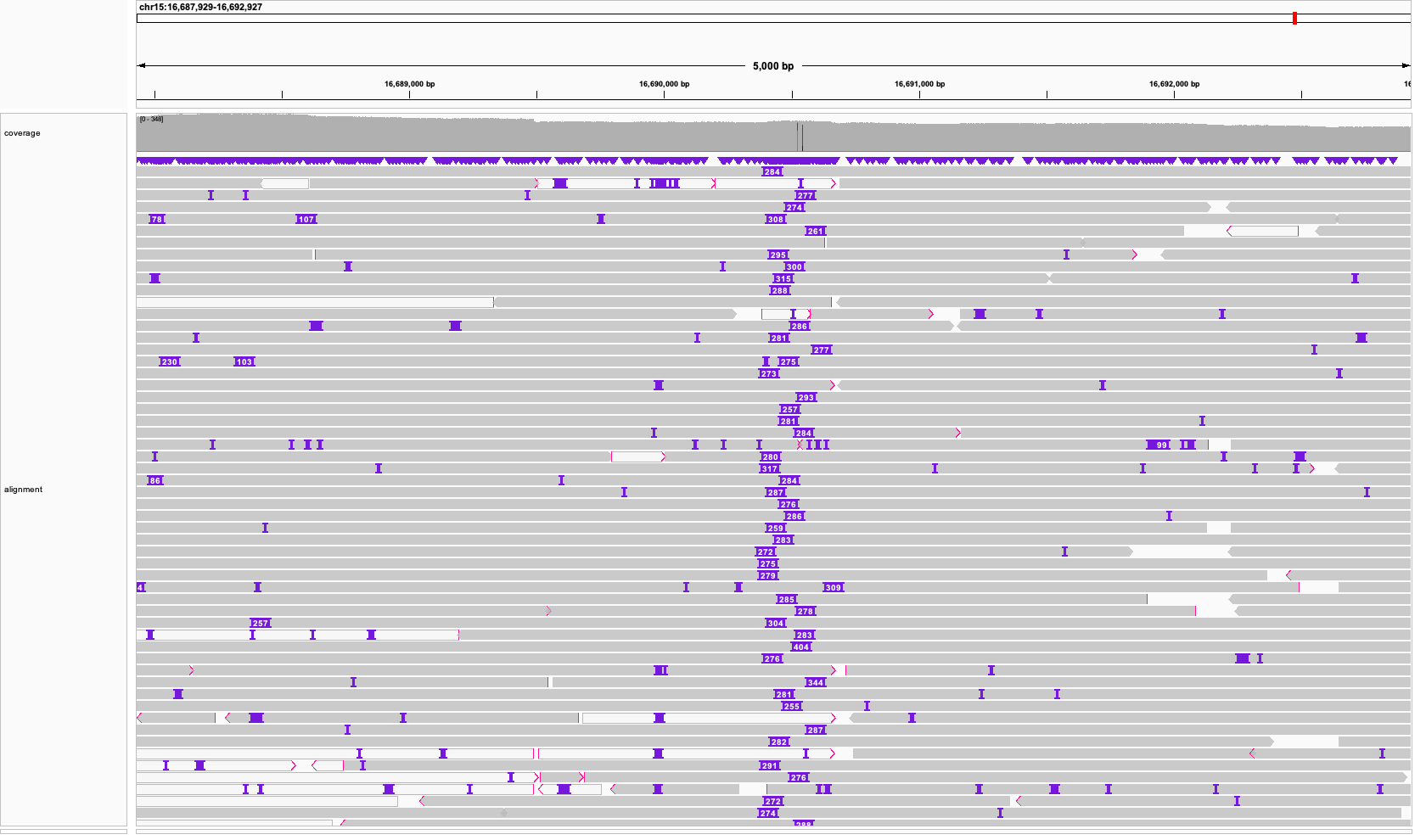

Supplement: jkad044_Supplementary_Data [file jkad044_supplementary_data.zip › Supplementary_Image/chr15.png]

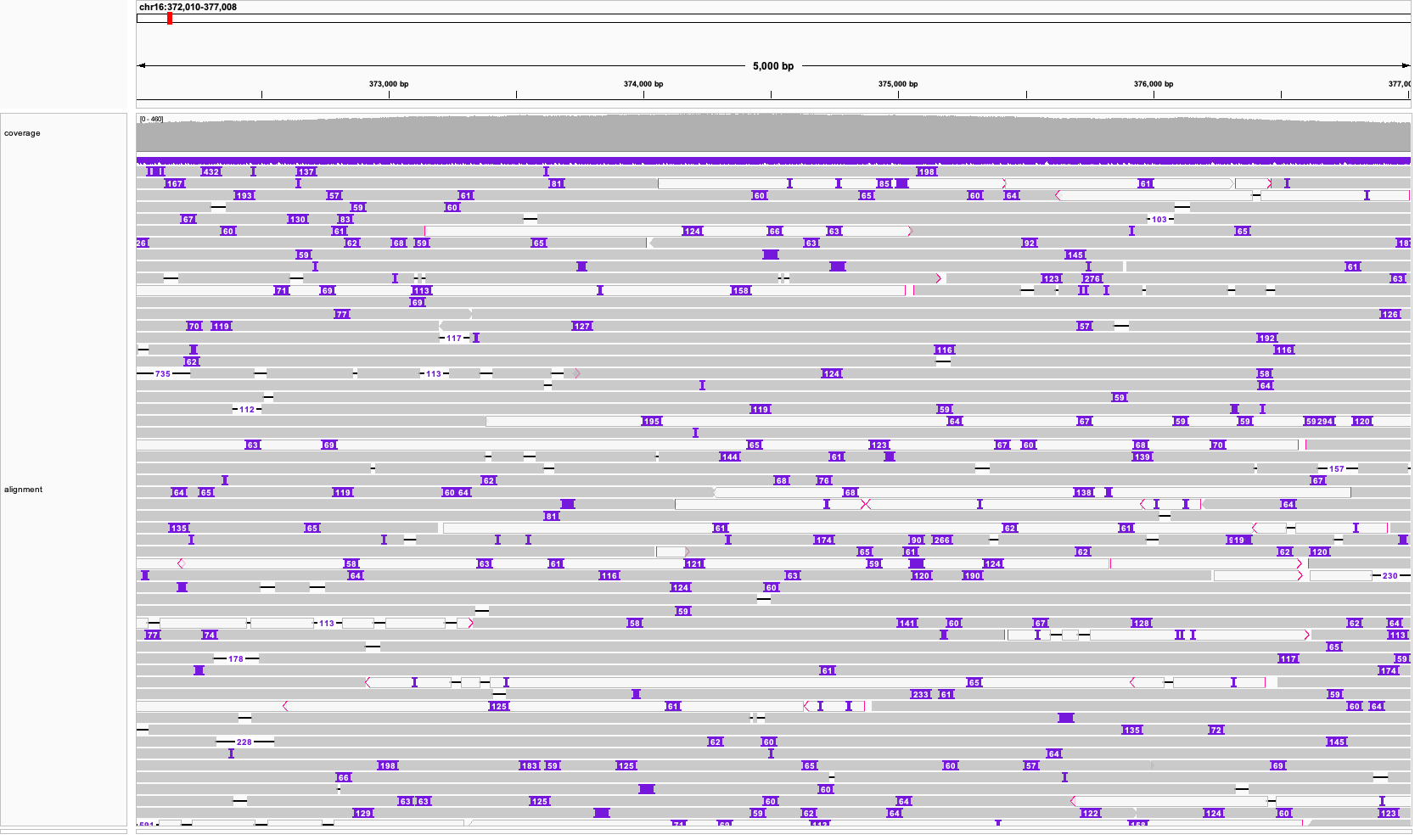

Supplement: jkad044_Supplementary_Data [file jkad044_supplementary_data.zip › Supplementary_Image/chr16.png]

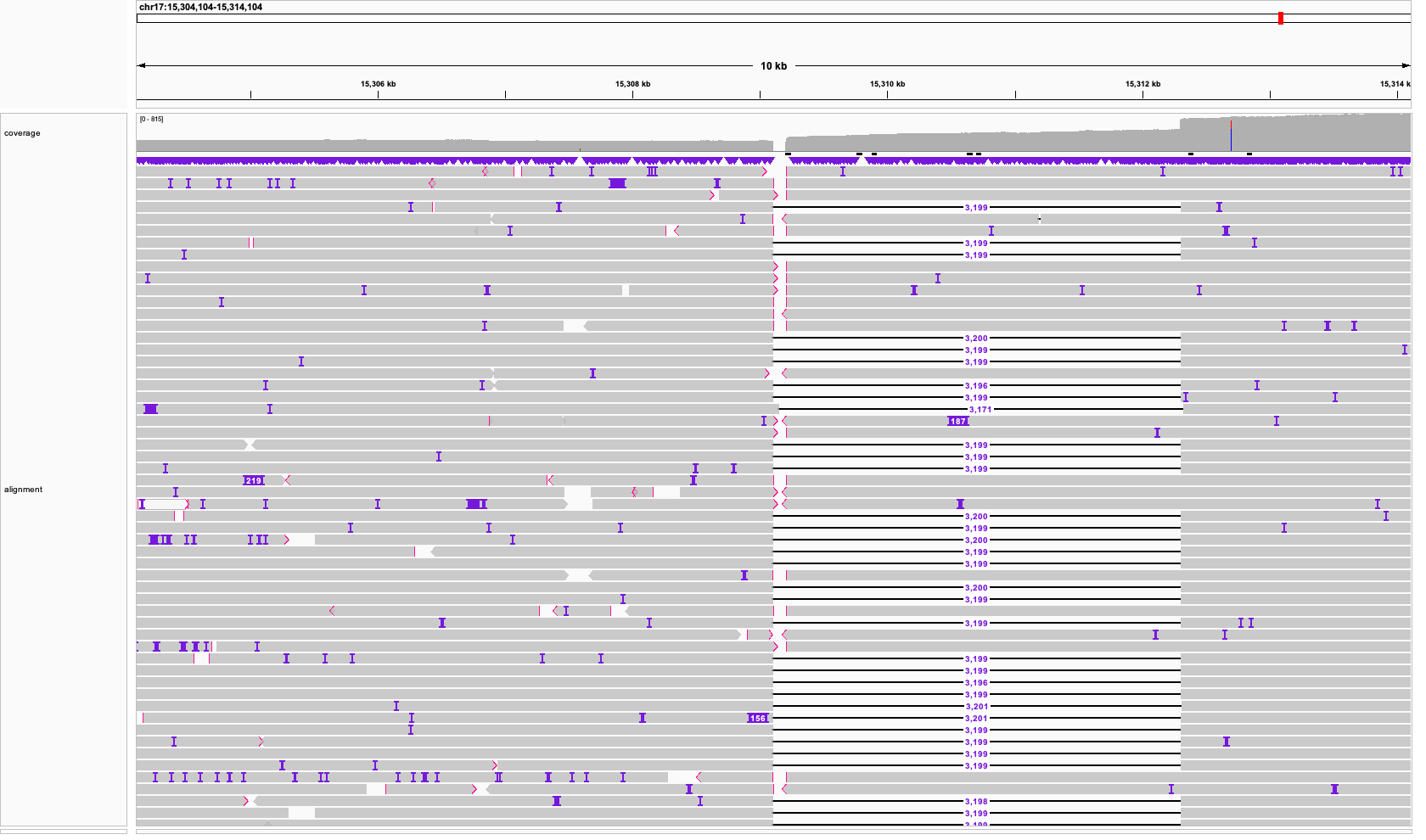

Supplement: jkad044_Supplementary_Data [file jkad044_supplementary_data.zip › Supplementary_Image/chr17.png]

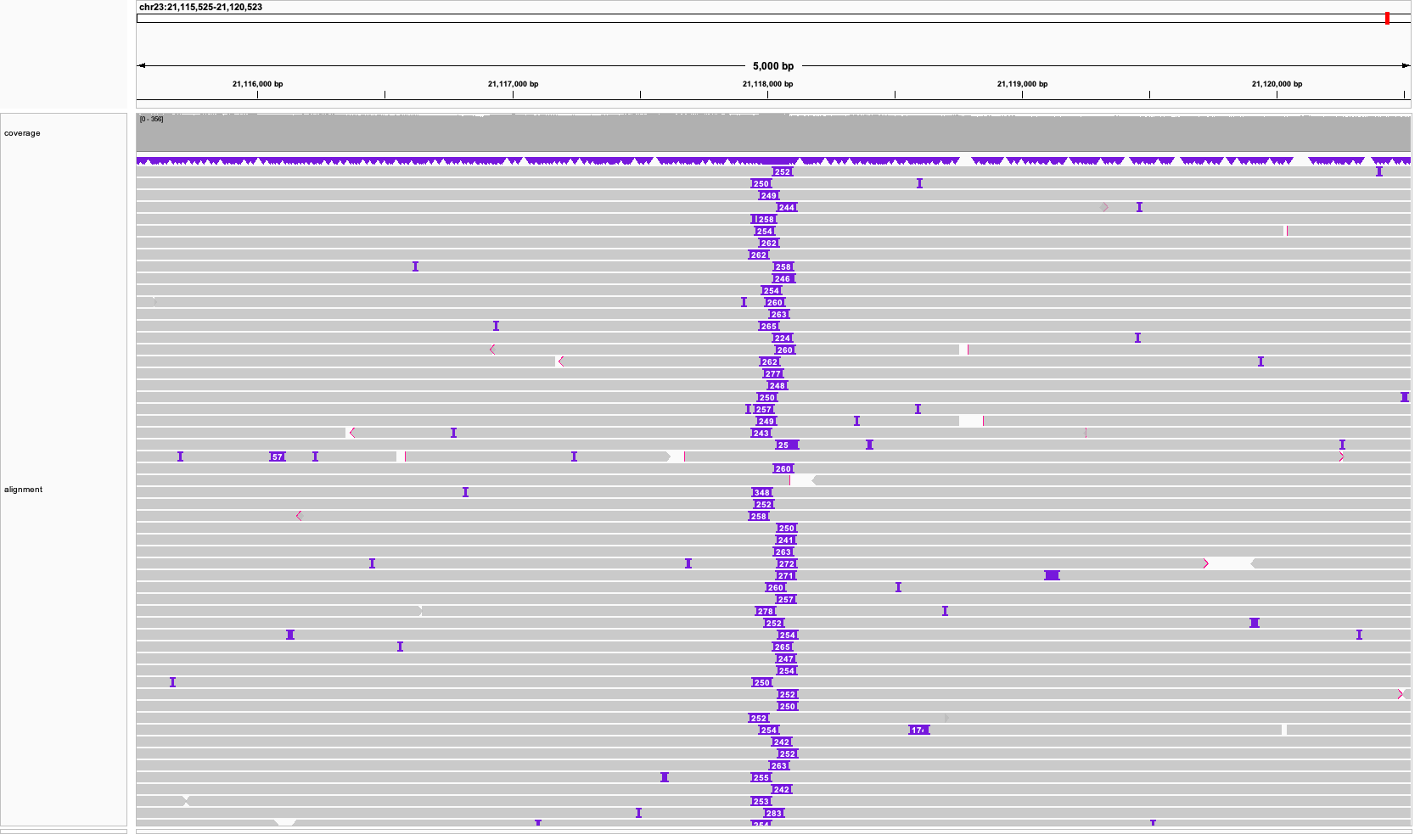

Supplement: jkad044_Supplementary_Data [file jkad044_supplementary_data.zip › Supplementary_Image/chr23.png]

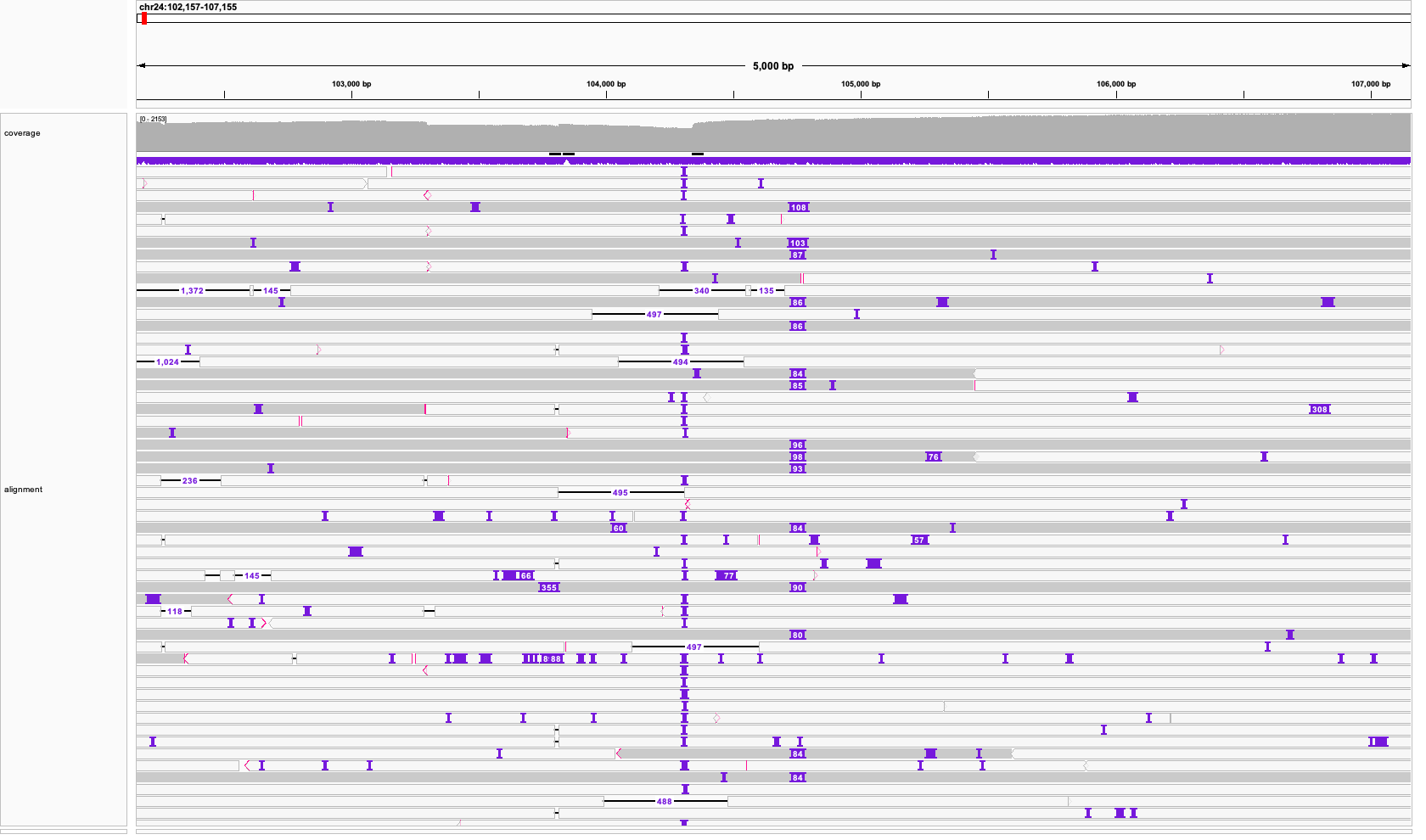

Supplement: jkad044_Supplementary_Data [file jkad044_supplementary_data.zip › Supplementary_Image/chr24.png]
